# A High-Intensity Exercise Intervention Improves Older Women Lumbar Spine and Distal Tibia Bone Microstructure and Function: A 20-Week Randomized Controlled Trial

**DOI:** 10.1109/JTEHM.2019.2963189

**Published:** 2020-01-03

**Authors:** João Pedro Pinho, Arturo Forner-Cordero, Rosa Maria Rodrigues Pereira, Arnaldo José Hernandez, Egídio Lima Dórea, Bruno Mezêncio, Liliam Takayama, Jackeline Couto Alvarenga, Júlio Cerca Serrão, Alberto Carlos Amadio

**Affiliations:** 1 Laboratory of Biomechanics, School of Physical Education and SportsUniversity of São Paulo 28133 São Paulo05508-220Brazil; 2 Biomechatronics LaboratoryEscola Politécnica of the University of São PauloSão Paulo05508-220Brazil; 3 Institute of Advanced Studies 243366 São Paulo05508-220Brazil; 4 Bone Metabolism Laboratory, Faculty of MedicineUniversity of São Paulo 28133 São Paulo05508-220Brazil; 5 Department of Orthopedics and Traumatology, Medical SchoolUniversity of São Paulo 28133 São Paulo05508-220Brazil; 6 University Hospital, University of São PauloSão Paulo05508-220Brazil

**Keywords:** Finite element analysis, HR-pQCT, plyometric training, power training, trabecular bone score

## Abstract

**Introduction:** The effects of ageing on bone can be mitigated with different types of physical training, such as power training. However, stimuli that combine increasing external and internal loads concomitantly may improve bone quality. The goal of this study was to assess the efficacy of a combined power and plyometric training on lumbar spine and distal tibia microstructure and function. **Methods:** 38 sedentary elderly women between 60 and 70 years were randomly allocated in experimental (N = 21) and control group (N = 17). The effects of the 20-week protocol on lumbar spine microstructure and tibia microstructure and function were assessed by trabecular bone score (TBS), high resolution peripheral quantitative computed tomography (HR-pQCT) and microfinite element analysis. **Results:** when compared to the effects found in the control group, the experimental group showed significant improvements in lumbar spine TBS (Hedges’ g = 0.77); and in distal tibia trabecular thickness (g = 0.82) and trabecular bone mineral density (g=0.63). **Conclusion:** our findings underscore the effectiveness of the proposed intervention, suggesting it as a new strategy to slow down and even reverse the structural and functional losses in the skeletal system due to ageing.

## Graphical abstract

We showed that the effects of ageing on bone can be mitigated with power and plyometric training. A 20-week training protocol was applied on a group of ladies between 60 and 70 years old. They showed significant improvements in lumbar spine TBS (Hedges' g

## I. Introduction

Hip and vertebral fractures are the most common bone injuries among the elderly populations [1], [2]; and while a deteriorated bone structure explains higher risk of fracture it seems that muscle loss plays also a key role [3], [4]. There seems to exist a link, not fully understood yet [5], between muscle and bone status. A linear relationship between sarcopenia and bone degradation helps to explain a higher chance of bone fracture [6]. Increasing physical inactivity in old age reduces the loads acting on the skeletal system [7]. These internal (joint reaction forces) and external loads (ground reaction forces) applied upon the senescent locomotor system are needed in order to ensure its functional homeostasis [8]. Therefore, physical exercise is probably the best non-pharmacological strategy to overcome the deterioration found in old age, providing means to attenuate or even reverse bone loss [7], along with other benefits such as cardio, balance, motor control or even self-confidence. In the search for the best mechanical stimulation, several training strategies have been proposed based on increasing internal, external forces or both concomitantly [9]–[12].

While weight-bearing endurance activities, such as walking, are inexpensive, easily available and widely used, they seem to offer a modest stimulus to bone mineral density, at least at the spine level [13]. Consequently, strength training emerged as one of the most successful strategies to induce osteogenesis [11]. Nevertheless, more recent approaches aimed at guaranteeing structural improvements at the musculoskeletal system along with higher functional capacity [14] while maintaining higher quality of life [15]. In addition to improve functional capacity, power training seems to be a better strategy to attenuate losses on the senescent skeletal system [16]. Stengel *et al.* [16] reported lower bone mineral density (BMD) losses at the lumbar spine on postmenopausal women after a two-year high velocity training than on the group that trained at moderate velocity. Since the training volume was the same in both groups, the authors reported that higher loading rates on the musculoskeletal system due to higher velocities could be the cause of these findings.

The stretch-shortening cycle potentiation of a muscle induces even higher loading rates on the musculo-skeletal system and it is widely used by athletes to increase muscle power [17]. This strategy allows augmenting the force production in the concentric phase of the movement by releasing previously stored elastic energy in the muscle [18]. The use of this elastic energy can be optimized by a high muscle preactivation followed by a rapid transition between eccentric and concentric phases of the movement [17]. Via plyometric training, an intervention methodology that induces this strategy, adult athletes showed increases in muscle power as well as in bone mass [19].

This strategy has already been applied with older adults; however, an issue arises concerning the instructions given to ensure that the potentiation mechanism was accurately employed. Indeed, while some authors refer solely to the training volume, such as number of jumps per session, others state that the jumps were performed quickly, neglecting the focus on the rapid eccentric-concentric transition [20], [21].

Another limitation to evaluate the effect of any intervention strategy on the bone is the instrument resolution. Cancellous bone has a higher bone turnover rate than cortical one [22]; therefore, it is expected that the mechanical load from training induces different stimulus on these bone structures. Indeed, Hamilton *et al.* [23] reported in a review different responses to exercise training on the bone microstructure of elderly volunteers. While only half of the studies found positive effects of the intervention on trabecular bone, the majority found positive effects on cortical bone. The authors concluded that to assess the bone response to an intervention, it is necessary to measure both cortical and trabecular structures [24].

In addition, it can be agreed that the main goal of an intervention protocol is to increase the load on the bone safely and improve its functionality; however, mores studies are needed to evaluate the effects of a physical intervention program on bone function. The reported effects of physical exercise protocols on bone are limited to structural or microstructural analysis [9]–[12]; and the meaning of a change in this structure for functional purposes is not clear [25]. Therefore, it is crucial to measure bone function in order to assess the validity of an exercise intervention program on elderly people.

Finally, an important aspect is to identify the minimum intervention time. The effect of a 19-week exercise program on the distal tibia trabecular bone density of elderly stroke survivors was reported in Pang *et al.* [26]. While other studies consider implicitly that a minimum of 24 weeks is required to determine effects on bone from physical exercise [10], this assumption can be revised in the light of more accurate results provided by new experimental procedures, including measurement devices with higher resolution or intervention techniques.

We hypothesize that the combination of power and plyometric training can be very efficient to induce changes on the elderly bone, even in relatively short time spans.

The main goal of this work is to investigate the effects of a 20-week exercise program, based in the combination of power and plyometric training, on lumbar spine and tibia bone microstructure and function. Additionally, it will be checked if this intervention time is enough to induce measurable changes in bone structure and function.

## II. Methods

### A. Study Design and Participants

This was a randomized controlled trial with a parallel-group study design to assess the effects of 20 weeks of high impact exercises and power training on lumbar spine and distal tibia bone microstructure and function in older women.

The sample size was estimated from a previous study on the distal tibia trabecular bone density (standardized effect size=0.9) found in Pang *et al.* [26]. Therefore, with a 5% significant level and an expected power of 0.85 the required sample size was 28, 14 for each group. Considering possible dropouts, the experimental group (EG) was increased by 50% while the control group (CG) was increased by 20%. Therefore, thirty-eight elderly women volunteered to participate in this study, and they were randomly allocated to the experimental group N = 21(66.9 ± 4.2 years) and N = 17 in the control group (65.0 ± 3.4 years). The inclusion criteria were: women between 60 and 70 years old, absence of cardiovascular, osteoarticular, musculoskeletal or neurological disorders, uncompensated visual problems, depression or mental illness, negative history of falling or dizziness during one year prior to the study, absence of osteometabolic diseases (such as hyperparathyroidism) or chronic diseases (diabetes mellitus, kidney or liver failure, hyperthyroidism). They were not using medication that may interfere with bone metabolism (such as bisphosphonates, teriparatide, glucocorticoids).

The participants were considered sedentary or participating, at most, in sporadic aerobic physical activities (maximum biweekly frequency). Attending less than 75% of the exercise sessions (for the EG) and the absence in the final evaluation (for both groups) were defined as the exclusion criteria. This study was registered in ClinicalTrials.gov and attends CONSORT 2010 statement.

All participants were informed about the experimental procedures and gave their signed consent informing their involvement in the study was voluntary. The study was approved by the Local Ethical Committee.

### B. Training Protocol

Thrice-weekly sessions of 60 minutes in non-consecutive days for 20 weeks were applied to the EG. The training session was divided into a main part (55 minutes) and a cool down (with stretching exercises). Fourteen exercise stations were applied: drop jump (2 stations), squat jump, leg press, knee extension, knee flexion, ankle dorsiflexion in a low pulley, body weight ankle plantarflexion, chest press, seated row, abdominal muscles exercise and resting (3 stations). Thus, the jump stations had a focus on the external load applied to the musculoskeletal system while the power exercises had a focus on the internal load on the bone. Three sets of 10 repetitions in each station were executed before station rotation; and, at every three exercises, a resting station was given. To avoid order influence, the order of the stations was changed every session and high physical demand exercises (jump stations) were distributed over the training session.

The two initial weeks were used as a familiarization period with the training. At week 3, and every four weeks after that, Brzycki [27] estimation of 1 repetition maximum (1RM) was obtained for each exercise and the load intensity for lower (50% of 1RM) and upper limbs (60% of 1RM) were established. For the drop jump exercise, a step with 9 cm height was used (for the first 6 weeks) that later was replaced by a step with 18 cm height.

A 1:2 instructor/participant ratio was needed to ensure the exercises were executed with the fastest concentric phase or eccentric/concentric transitions. Instructions were constantly given to maximize power output, except for upper limbs and abdominal exercises that were asked to be executed at moderate speed.

Participants in both groups were evaluated before and after the intervention period. The modified Baecke questionnaire for older adults [28] was used to assess the participants physical activity level. Borg’s perceived exertion scale [29], given at the end of each session, was used to characterize the intervention protocol. In order to characterize the jumps intensity, a force plate (AMTI BP600900), with a sampling rate of 200 Hz, was used. After the intervention period, the EG participants performed six repetitions of the drop jump (3 with 9 cm and 3 with 18 cm) and three repetitions of the squat jump on a force plate.

### C. Lumbar Spine Microstructure

All DXA measurements as well as the trabecular bone score (TBS) analysis, prior and after the intervention period, were performed by the same experienced operator. Total lumbar spine (L1-L4) areal BMD (aBMD) was assessed with a bone densitometer (Hologic Inc. Bedford, MA, USA, Discovery model). The DXA scans were assessed according to the International Society for Clinical Densitometry guidelines [30].

To calculate lumbar spine TBS a software by TBS iNsightⓇ, version 2.2.0.1 (Med-Imaps, Merignac, France), was used. The TBS software takes into account the pixel gray-level variation in the total lumbar spine aBMD image so that low/large number of pixel value variation of high/small amplitude indicates a 2D projection of a deteriorated/good trabecular structure. This new method to describe skeletal microarchitecture from DXA images is strongly correlated with bone histomorphometry [31], [32]. Indeed, it is correlated with micro-computed tomography measures of bone connectivity density, trabecular number, trabecular separation and with vertebral mechanical function. Therefore, low/high TBS values are correlated with worse/better trabecular bone structure.

### D. Distal Tibia Microstructure and Function: Bone Morphometry and Tissue Mineral Density

All microarchitecture and function analysis, prior and after the intervention period, were performed by the same experienced operator. Distal tibia microarchitecture and volumetric BMD (vBMD) were assessed with a high-resolution peripheral quantitative computed tomography (HR-pQCT) system (Xtreme CT Scanco Medical AG, Brüttisellen, Switzerland) on the dominant limb. The 2D detector array in combination with a 0.08 mm point-focus x-ray tube allowed an acquisition of 110 CT slices with a nominal resolution (voxel size) of 82 $\mu \text{m}$, providing a 3D representation of approximately 9 mm of the distal tibia. The settings used were: effective energy of 60 kVp, x-ray tube current of 95 mA, and matrix size of $1536\times1536$.

The participants were asked to seat comfortably with the dominant leg immobilized with a carbon fiber cast. Verbal cues were given to avoid movement artifacts.

The operator defined a reference line at the endplate of the tibia and the first CT slice taken was 22.5 mm proximal to the reference line. Using a threshold-based algorithm, the entire volume of interest was separated into cortical and trabecular regions. One third of the apparent cortical bone density value (Ct.BMD) was used to discriminate cortical from trabecular region. The microstructure parameters used were: 1. vBMD in milligram hydroxyapatite per cubic centimeter (mg HA/cm^3^) for total (Tt.BMD), trabecular (Tb.BMD) and cortical (Ct.BMD) regions; 2. trabecular microstructure parameters: bone volume fraction (BV/TV, 1), thickness (Tb.Th, mm), number (Tb.N,mm^−1^) and separation (Tb.Sp, mm); and 3. cortical microstructure parameters: thickness (Ct.Th, mm), porosity (Ct.Po, 1) and mean pore diameter (Ct.Po.Dm, mm^2^).

### E. Microfinite Element Analysis

Microfinite element model to represent distal tibia biomechanical properties were created in Scanco Finite Element Analysis Software (Scanco Medical AG), using the distal tibia HR-pQCT scan. It was defined a Young’s modulus of 10 GPa and Poisson’s ratio of 0.3 for each element. Bone strength (i.e., failure load), based on biomechanical properties, was derived by scaling the resulting load from a test simulating 1% compression, such that 2% of all elements had an effective strain >7000 microstrain. The following structural functional parameters were used: stiffness (S, N/mm), estimated ultimate failure load (F. ult, N), trabecular and cortical von Mises stress (Tb.VM and C.VM, respectively, N/mm2).

### F. Statistical Analysis

All statistical procedures were executed in Statistical Package for the Social Sciences (SPSS for Windows, 20.0, Chicago, IL, USA). A visual inspection of the data was firstly performed in order to identify outliers. Then, Shapiro-Wilk test was used to assess the data distribution; and Levene test was used to assess the data cedasticity. Since all dependent variables exhibited normal distribution, multivariable normality was assumed. Box’s M was used to test the equality of the variance/covariance matrices.

An unpaired t-test was used to identify differences, prior intervention, between groups on age, mass, height, physical activity level, femoral neck T-score, lumbar spine T-score and in all bone microstructure and function dependent variables. To compare the effects of the intervention period between groups, a general linear mixed effect model was conducted. Time and Group was assumed to be a fixed effect in the model with the participants as a random effect. The magnitude of the intervention period effects between groups was determine by Hedges’ g effect size and respective 95% confidence interval [35]. Cohen’s effect size benchmark [36] of trivial (−0.2≤d≤0.2), small (−0.5≤d<−0.2 and 0.2<d≤0.5), moderate (−0.8≤d<−0.5 and 0.5<d≤0.8) and large (d<−0.8 and d<0.8) was employed.

## III. Results

A total of 353 older women reply to the public advertising and after the medical report analysis and the interview 43 attended all requirements. Due to schedule-related incompatibility, the final sample was composed by 38 participants. The participants in the EG attended, on average, to 92% of the planned training sessions and none attended less than 80%. No EG nor CG participants met the exclusion criteria, therefore, the study had no dropouts.

### A. Participants Characteristics at Baseline

Initial assessment of both groups prior the intervention period showed no differences on age, anthropometric characteristics, physical activity level nor on femur and lumbar spine bone density (Table 1).

**TABLE 1:** Participants Characteristics at Baseline

	EG (n=21)	CG (n=17)	t	P
Age, years	66.9 (65.0-68.8)	65 (63.3-66.7)	1.507	0.141
Mass, Kg	65.5 (62.1-68.9)	70.3 (65.7-74.9)	−1.805	0.079
Height, m	1.60 (1.50-1.60)	1.60 (1.50-1.60)	−0.471	0.640
Physical Activity Level	8.9 (6.9-10.9)	9.6 (6.7-12.5)	−0.428	0.672
Femoral Neck T-score	−0.92 (−1.38–−0.46)	−1.35 (−1.66–−1.04)	1.558	0.129
Lumbar Spine T-score	−1.42 (−1.69–−1.15)	−1.37 (−1.73–−1.01)	−0.237	0.814

Values expressed in mean and 95% confidence interval (lower– upper). EG: experimental group. CG: control group. t: independent T test t-value. P: independent t test P-value.

All bone microstructure and function dependent variables were tested at baseline and no differences were found between the EG and the CG (P-values for the independent t tests ranged between 0.218 and 0.882). All variables showed a significant pre/post intervention correlation (Pearson’s r ranging from 0.995 to 0.665, with P-values ranging from < 0.001 to 0.005), allowing the inclusion of the pre-intervention value as a covariable. It must be noted that both groups were found to be, at the beginning of the study, within the same TBS category – partial degraded microarchitecture (TBS≤1.2 defines degraded microarchitecture, TBS between 1.20 and 1.35 is partially degraded microarchitecture, and TBS ≥1.35 is considered normal) [37]–[40].

### B. Training Intensity

The mean perceived exertion measured after the training session was 9.5 (9.3 – 9.7), for all training sessions in the 20-week period. In the first training stage of the drop jump (9cm step) the participants produced an impact of 2.4 (2.2 – 2.6) BW (body weight) and in the second (18cm step) an impact of 3.3 (2.9 – 3.7) BW. In the squat jump exercise a mean impact of 4.0 (3.7 – 4.3) BW was produced with a mean height of 9.7 (8.4 – 11.0) cm.

### C. Outcome Measures

**FIGURE 1.**
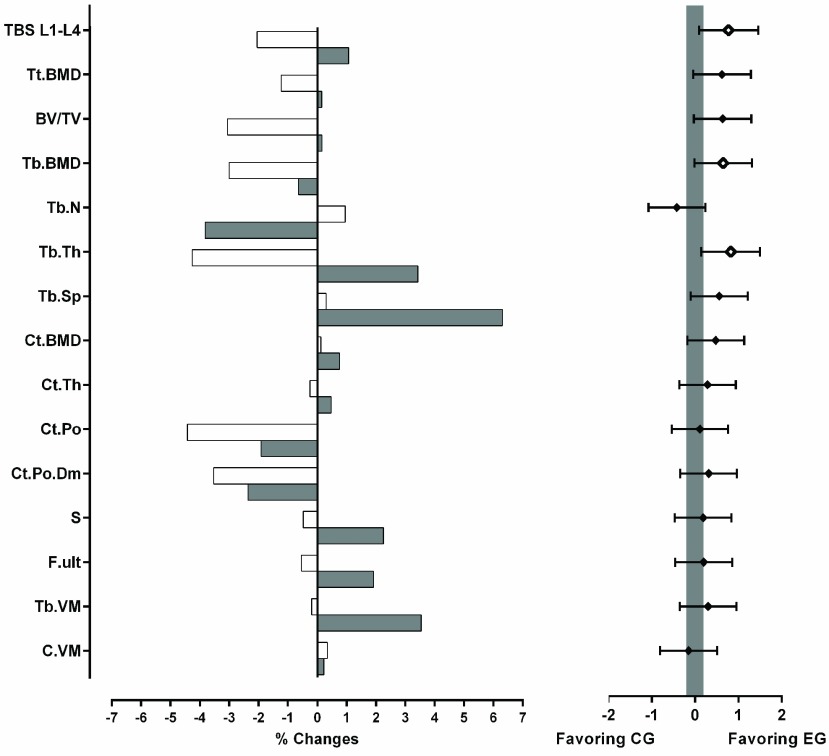
Mean percentage change after the intervention period in the experimental group (shaded bars on the left graph) and in the control group (white bars on the left graph) of lumbar spine (L1-L4) trabecular bone score (TBS L1-L4) and tibial microstructure and function. BMD: bone mineral density. BV: bone volume. TV: total volume. Tb: trabecular. Ct and C: cortical. Tt: total. Tb.N: trabecular number. Th: thickness. Tb.Sp: trabecular separation. Po: porosity. Po.Dm: mean pore diameter. S: stiffness. F. ult: estimated ultimate failure load. N: Newton. VM: von Mises yield criterion. The graph on the right expresses Hedges’ g effect size and 95% confidence intervals. A blank mark denotes significant difference (p < 0.05) between the two groups and a solid mark the absence of significant differences. The shaded area specifies the interval in which the effect size of the difference between groups is trivial (−0.2 < g < 0.2).

Significant changes were found between groups after the intervention period solely for the bone trabecular structure and function (Table 2). While cortical bone seems to remain unchanged, an improvement was found in lumbar spine TBS and tibia trabecular thickness of the EG (Fig. 1).

**TABLE 2:** Comparison Between Groups of the Effects of the Intervention Period on Bone Microstructure and Function

	EG (n=21)		CG (n=17)		P
	Pre-Intervention	Post-intervention	Pre-Intervention	Post-intervention	
Lumbar spine TBS	1.260 (1.224-1.296)	1.274(1.227-1.321)	1.226 (1.180-1.272)	1.201 (1.153 -1.249)	0.025
Tt.BMD (mg HA/cm3)	268.1 (243.9-292.3)	268.5(243.4-293.7)	252.6(223.1-282.2)	249.5(219.3-279.8)	0.101
Tb.BMD (mg HA/cm3)	144.2 (126.1 - 162.4)	143.3 (124.1 - 162.5)	129.9 (112.1-147.6)	126.0 (107.8 -144.2)	0.032
BV/TV(1)	0.120(0.105-0.135)	0.120(0.104-0.136)	0.108(0.093 - 0.123)	0.105(0.090 - 0.120)	0.093
Tb.N (mm-1)	1.684(1.547-1.821)	1.620(1.464-1.776)	1.638 (1.473 - 1.803)	1.653 (1.478-1.828)	0.294
Tb.Th (mm)	0.071 (0.065 - 0.077)	0.073 (0.067 - 0.079)	0.066(0.059 - 0.073)	0.063 (0.056 - 0.070)	0.027
Tb.Sp (mm)	0.543(0.482-0.604)	0.577 (0.502 - 0.652)	0.572 (0.491 - 0.653)	0.574 (0.482 - 0.666)	0.237
Ct.BMD (mg HA/cm3)	847.9(821.8-874.0)	854.3(826.8-881.9)	849.5 (822.1 - 876.9)	850.5 (821.3 - 879.7)	0.394
Ct.Th (mm)	1.012(0.910-1.114)	1.017(0.915-1.119)	1.016(0.904-1.128)	1.014(0.897-1.131)	0.403
Ct.Po (%)	0.065(0.054-0.076)	0.064(0.052 - 0.076)	0.065 (0.051 - 0.079)	0.062 (0.049 - 0.075)	0.824
Ct.Po.Dm (mm2)	0.186(0.175-0.197)	0.182(0.172-0.192)	0.190(0.179-0.201)	0.183(0.172-0.194)	0.574
S (N/mm)	176934 (162545-191323)	180914 (166165-195663)	173707(160636-186778)	172874(160261 -185487)	0.130
F. ult(N)	8454(7798-9110)	8617(7947-9287)	8326(7732 - 8920)	8281 (7709 - 8853)	0.980
Tb.VM (N/mm2)	54.2(50.9-57.4)	56.1 (52.6-59.5)	52.1 (49.0-55.2)	52.0(48.9-55.1)	0.239
C.VM (N/mm2)	87.4 (86.8-88.1)	87.6 (86.9 - 88.3)	86.7 (85.7 - 87.8)	87.0 (86.2 - 87.9)	0.684

Values expressed in mean and 95% confidence interval (lower - upper). EG: experimental group.CG: control group. P: Interaction effect in the mixed model P- value. TBS: trabecular bone score. BMD: bone mineral density. BV: bone volume. TV: total volume. Tb: trabecular. Ct and C: cortical. Tt: total. Tb.N: trabecular number. Th: thickness. Tb.Sp: trabecular separation. Po: porosity. Po.Dm: mean pore diameter. S: stiffness. F. ult: estimated ultimate failure load. N: Newton. VM: von Mises yield criterion.

## IV. Discussion

Our findings support that the proposed physical exercise protocol is a viable tool to reverse bone losses in osteopenic [41] elderly women with low to moderate physical activity level [28], even at short intervention periods (less than 6 months). Moreover, this is the first study to show the effects of an exercise program on lumbar spine microstructure via trabecular bone score as well as its effects on distal tibia bone function via microfinite element analysis.

The combination of impact exercises, that increase external loads of the skeletal system, with strength training, that increase internal loads, has been suggested as an optimal protocol to improve BMD in postmenopausal women [42]. Our results support and extend this recommendation. Activities focused on increasing muscle contraction forces, such as in hypogravity environments (e.g., swimming or cycling) should produce high forces on bone [43]; however, they did not induce bone structural or functional gains in older adults according to previous studies [44], [45]. However, internal loads seem to amplify the effects of the external forces (i.e., ground reaction forces) [44] resulting that a combination of internal and external loads seems to be the best strategy to improve a senescent bone. Our findings support the efficacy of this strategy on elderly women bone microstructure with a low perception of physical demand – 9.5 on Borg’s [29] perceived exertion scale (between the descriptors “Very light” and “Fairly light”).

Although we found significant effects of this intervention on tibia trabecular bone mineral density, the same effects were not seen in the cortical portion. Similar results on tibia cortical vBMD (−0.06%) were obtained by Liu-Ambrose et al. [46] after a 25-week strength training protocol, however, a second experimental group (agility) revealed an expressive increase of 5.32% [46]. Nonetheless, the use of bisphosphonate therapy on participants was not controlled, and this is a confounding factor that did not allow attributing the bone changes to the intervention program. Karinkanta *et al.* [47], in turn, found no significant nor clinical changes on elderly women distal tibia trabecular (0.00%) and cortical (−0.17%) vBMD after a 12-month combined resistance and balance-jumping training. Although our protocol did not show significant increases in distal tibial vBMD (total, trabecular nor cortical), the moderate effect sizes obtained (0.62, 0.65 and 0.47, respectively) suggest its efficacy in the decrease of vBMD losses (Fig. 1).

Paradoxically, tibia trabecular bone changes induced by the intervention period in the EG expressed a decreased number of trabeculae and an increased trabecular separation, suggesting a tissue deterioration. Since both parameters were preserved in the CG, a possible explanation is that thinner trabeculae were resorbed due to increased mechanical stimulus, reducing its number [48], [49], augmenting its thickness. Thinner trabeculae might be susceptible to resorption induced by mechanical stimuli, which contribute to thicker trabeculae and a wide space between the remaining trabeculae [48], [49]. Nonetheless, the bone volume fraction preservation (0.16%) suggests no harmful changes, corroborated also by an increasing ability to resist loads shown in the finite element analysis. Indeed, tibia stiffness and von Mises stress values significantly increased after the intervention period, indicating an increased resistance of the bone structure. This microfinite element analysis provides a direct estimate of the bone mechanical properties and indicates an improvement at a microstructural level consistent with a bone tissue more resistant to fractures [50]. Although the change in the stiffness represents an enhancement of the bone tissue, the change in von Mises stress seems to have more important implications, since it represents the trabecular bone increased ability to endure forces in different directions. Hence, since the forces acting on the musculoskeletal system are three-dimensional [51], this parameter offers higher ecological validity to understand the functional effects of an intervention.

Karinkanta *et al.* [47] found a decrease (−1.20%) in bone strength index with a combined resistance and balance-jumping protocol. Since the control group experienced a higher reduction on this parameter (−2.93%), the authors argued that the proposed protocol was able to attenuate the losses on bone strength [47]. Our findings, however, support that the presented intervention protocol enhances bone strength. Similarly, Allison *et al.* [52] found significant improvement in femoral neck biomechanical variables after 12 months of a home-based impact exercise intervention in elderly men. Unilateral jumps that elicited a load of 2.7 to 3.0 BW were found to increase femoral neck cross-sectional moment of inertia (2.4%) and decrease its buckling ratio (−8.3%), in the exercise leg, but also in the control leg, (0.9% and −4.6%, respectively); suggesting interference between them [52]. Nonetheless, it was a one-year daily intervention with twice the mechanical load offered in the present study.

We obtained positive results in only 20 weeks with half the load, indicating a continuous improvement during the intervention period. Ashe *et al.* [53], showed no differences after 12 months of strength training (biweekly sessions) in tibia cortical volumetric bone mineral density (−0.45%) nor in bone strength (0.05%) assessed by cross-sectional moment of inertia.

Due to lack of similar methodologic approaches, the positive effects of our high-intensity exercise intervention strategy can only be compared to the effects of a drug-therapy intervention. In a 12-month pharmacologic intervention in postmenopausal women, Tsai *et al.* [54] found that a combined teriparatide and denosumab therapy improved tibia stiffness and failure load by 5.3% and 4.5%, respectively. Although these responses are superior to those found in the present study (2.25% and 1.92%, respectively), they were reached achieved after an much longer intervention and using drugs that could induce serious side effects such as atrial fibrillation [55] or esophageal cancer [56]. Furthermore, it has also been reported that pharmacological intervention may have poor compliance [57] either due to unintentional (forgetfulness) or intentional nonadherence (due to treatment costs and fear of side effects). Moreover, a physical exercise intervention such as the proposed in our work has positive side-effects, as it not only promotes bone gains but also improves musculoskeletal system functional capacity.

We found the loads applied by the intervention protocol suitable to induce changes in the lumbar spine microstructure of osteopenic elderly women. While the changes in the EG (1.07 %) allowed approaching the TBS upper bound (1.300 – “good microarchitecture”), the absence of stimulus in the CG induced a decrease in TBS (−2.05 %), reaching the lower threshold (1.200 – “degraded microarchitecture”) [37]. This outcome suggests a significant decrease of risk fracture induced by the intervention protocol that, due to the lack of studies with similar approach, can only be compared with the effects of a pharmacologic intervention.

A physical exercise program seems to be superior since not only improves bone status but contributes to increasing functional capacity and quality of life [58]. Furthermore, it can be argued that the effects of pharmacologic intervention might be overestimated in the elderly population. For instance, Krieg *et al.* [59] found an annual increase of 0.20% in older women TBS with different bisphosphonates drugs. Therefore, the exercise-based intervention we presented could be a first line of defense against bone deterioration before the pharmacological treatment that has also showed significant impact on bone architecture [54], [60], [61]. It is very likely that the combination of different treatments, (exercise, pharmacological) with individualized planning for each patient, would produce better results. In a cross-sectional study [62], Heiniö and colleagues showed that female athletes that underwent different types of physical activity had small differences in TBS score. The authors suggested low impact exercises (like walking or endurance running) may lead to lower TBS scores when compared to high impact exercise, such the type of exercise that we proposed in this work.

There is a general consensus about the health benefits of exercise, that can be recommended as a prescription [63], [64]. In this respect, the set of exercises recommended showed clear improvements in bone function and they could be easily integrated in a more complete exercise program involving cardio-pulmonary, motor control and balance training. Our results support the hypothesis that continuing the proposed intervention for longer periods may delay the natural ageing bone decay and potentially increase bone health and this points out a line of future research. The investigation of the mechanisms by which the intervention program improved the participants’ bone health was not our main goal. However, it was considered that the compression forces produced by the muscles and the impacts, which are enhanced by the proposed training method, would explain our results. The impact, more specifically the strain energy density on trabecular bone elicited by the intervention, agree with the classic models of bone formation [43], [65], [66].

The results of the present study must consider some limitations. Hip fracture is probably the biggest problem in elderly population [67]. However, we were not able to assess the efficacy of this intervention protocol on this bone site. Moreover, caution must be taken when prescribing any exercise protocol to frail older adults due to the risk of falls and because the loads applied by the physical activity, and even more jump exercises may be too high for a deteriorated bone structure and lead to a fracture. Although another limitation of this work is that we could not apply the finite element modeling to lumbar spine, TBS is found to be intimately related with the tissue microstructure and with its function [68]. Indeed, it is correlated to the number of trabeculae, its separation and connectivity density; that represents a fracture resistant or prone micro-architecture yielded by higher or lower scores, respectively [69].

## V. Conclusion

In conclusion, we found the 20-week power/plyometric training protocol able to improve tibial bone age-related microstructure degradation enhancing its functional stiffness and resistance to fracture in elderly women. Moreover, the presented high-intensity exercise intervention was able to induce changes in lumbar spine microarchitecture consistent with a more fracture-resistant status. Considering its high adherence could be a fundamental part of a non-pharmacologic strategy to reverse bone loss in older adults.
